# Comparison of fibrosing mediastinitis patients with vs. without markedly increased systolic pulmonary arterial pressure: a single-center retrospective study

**DOI:** 10.1186/s12872-022-02567-z

**Published:** 2022-03-31

**Authors:** Xinyuan Zhang, Shu Zhang, Jianfeng Wang, Wei Jiang, Lanlan Sun, Yuanzhi Li, Dichen Guo, Yuanhua Yang, Xiuzhang Lu, Yidan Li

**Affiliations:** 1 Department of Echocardiography, Heart Center, Beijing Chao-Yang Hospital, Capital Medical University, 8 Gongren Tiyuchang Nanlu, Chaoyang District, Beijing, 100020 China; 2 Department of Respiratory and Critical Care Medicine, Beijing Chao-Yang Hospital, Capital Medical University, Beijing, 100020 China; 3 Department of Intervention, Beijing Chao-Yang Hospital, Capital Medical University, Beijing, 100020 China

**Keywords:** Fibrosing mediastinitis, Pulmonary hypertension, Echocardiography, Balloon pulmonary angioplasty, Retrospective cohort

## Abstract

**Introduction:**

Fibrosing mediastinitis (FM) complicated with pulmonary hypertension (PH) has been considered as an important cause of morbidity and mortality. This study was designed to observe the possible effects of abnormal hemodynamics on patients by conducting a between-group comparison according to the presence of markedly increased systolic pulmonary arterial pressure (SPAP), so as to provide more information for clinical management.

**Materials and methods:**

Fifty-one patients with clinically diagnosed FM were divided in two groups (SPAP < 50 mmHg group; SPAP ≥ 50 mmHg group) and retrospectively included in the study. Data mainly including demographic factors, echocardiographic data, results of right heart catheter and computed tomography (CT) examination were retrieved from the medical database. Echocardiographic parameters pre- and post- balloon pulmonary angioplasty (BPA) treatment were also collected in 8 patients.

**Results:**

Significant changes in cardiac structure, hemodynamics and cardiac function were detected in patients complicated with markedly increased SPAP. Patients in the SPAP ≥ 50 mmHg group had increased right heart diameter, right heart ratio and velocity of tricuspid regurgitation (VTR) (*p* < 0.05). Deteriorated right heart function was also observed. There was no significant difference in CT findings between the two groups, except that more patients in the SPAP ≥ 50 mmHg group had pleural effusion (*p* < 0.05). After primary BPA in 8 patients, improvement in the right atrium proportion was observed.

**Conclusions:**

Changes due to significantly increased SPAP in patients with FM include adverse structure and function of the right heart, but differences in CT findings were not significant. Echocardiography has advantages as a noninvasive tool for the evaluation of cardiac structure, function and hemodynamics in patients with FM.

**Supplementary Information:**

The online version contains supplementary material available at 10.1186/s12872-022-02567-z.

## Introduction

Fibrosing mediastinitis (FM) is an uncommon and progressive condition characterized by an invasive proliferation of fibrous tissue within the mediastinum. The condition is also referred to as “mediastinal fibrosis” or “sclerosing mediastinitis”. Although considered to be a benign disease, patients with FM often show compression and occlusion of mediastinal structures, such as the tracheobronchial tree, pulmonary vessels, and esophagus [1]. Patients with FM are likely to present various clinical manifestations and imaging findings, depending on the individual’s pathogenesis and the structures involved. To date, known causes of FM include histoplasmosis, tuberculosis, sarcoidosis, other granulomatous diseases, and radiation therapy [2–4]. However, the early diagnosis of FM is usually difficult, which is a primary reason for inadequate treatment of the disease and poor patient prognosis.

Clinical diagnosis of FM mainly depends on the manifestations on contrast-enhanced high-resolution computed tomography (HRCT) of the chest or computed tomography pulmonary angiography (CTPA) [5]. For patients with FM, functional evaluations also include echocardiography, pulmonary function testing, laboratory examination, and 6-min walk distance (6MWD) [6]. The efficacies of medical therapy are limited, and evidence-based treatments are lacking. However, interventional or surgical treatments, such as balloon angioplasty and pulmonary arterial bypass surgery, are often indicated for those with cardiac or pulmonary functional impairment caused by severe stenosis [7–9].

Pulmonary hypertension (PH) is a common complication in FM, associated with poor prognosis due to the high incidence of pulmonary vessels and tracheal tree involvement [6]. Patients with FM complicated with PH are more likely to suffer from progressive hemodynamic disorders and right heart failure [10]. For those with suspected PH, right-sided heart catheterization (RHC) is the gold standard for diagnosis. However, because RHC is invasive, echocardiography is commonly performed to evaluate patients for PH in a noninvasive fashion. It has been confirmed that systolic pulmonary arterial pressure (SPAP) measured with echocardiography is significantly positively correlated with those obtained via RHC [11].

Therefore, in this study, we aimed to investigate the effect of elevated pulmonary arterial pressure in patients with FM by comparing the characteristics of groups with and without significantly elevated SPAP, which may be helpful to improve the clinical management of these patients.

## Materials and methods

### Study patients

A total of 54 patients with FM that were diagnosed and treated at Beijing Chao Yang Hospital (Beijing, China) between Oct. 2009 and Jul. 2019 were enrolled in the present study. The diagnosis of FM was established by characteristic imaging manifestations (an infiltrative mediastinal process associated with the invasion or obstruction of mediastinal structures). Among the patients, 3 were excluded due to the diagnosis of pulmonary embolism. The remaining 51 patients were confirmed to be free of any history of lung or mediastinal malignancies or prior mediastinal radiation therapy, and included in the study.

The present study was approved by the Ethics Committee of Beijing ChaoYang Hospital (Beijing, China). Informed consent was waived because this was a retrospective study.

### Clinical data

The clinical data retrospectively extracted from the clinical database included: age, sex, symptoms, etiological factors, smoking history, erythrocyte sedimentation rate (ESR), serum level of c-reactive protein (CRP), arterial blood gas analysis (the arterial partial pressure of oxygen [PaO2], the partial pressure of carbon dioxide [PaCO2], oxygen saturation [SaO2]), respiratory function (percentage of forced vital capacity compared to predicted value [FVC%pred], percentage of the forced expiratory volume in 1 s compared to predicted value [FEV1%pred], the ratio of forced vital capacity to forced expiratory volume in 1 s [FVC/FEV1], percentage of lung diffusing capacity for carbon monoxide compared to predicted value [DLCO%pred] and percentage of total lung capacity compared to predicted value [TLC%pred]). An obstructive pattern was defined as FEV1/FVC < 0.7. A restrictive pattern was defined as predicted TLC < 80%. A mixed pattern was defined as the concurrence of both of these results.

### Echocardiographic examination

The echocardiographic measurements obtained included the inner dimensions of the cardiac chamber (basal left/right ventricular linear dimension in four-chamber apical view, left/right atrium transverse dimension in four-chamber apical view), internal diameter of the inferior vena cava (D_SVC_), SPAP, left ventricular ejection fraction (LVEF), diameter of the main pulmonary artery (D_MPA_), and the velocity of tricuspid regurgitation (V_TR_). To evaluate right ventricular (RV) function, the RV index of myocardial performance (RIMP), tricuspid annular plane systolic excursion (TAPSE), or RV fractional area change (RVFAC) were also measured. Previous studies by our group showed that an echocardiography SPAP cut-off value of ≥ 50 mmHg had good sensitivity and specificity for PH [11]. Patients were therefore divided into two groups according to SPAP at diagnosis (SPAP < 50 mmHg group; SPAP ≥ 50 mmHg group). Serial echocardiographic measurements were obtained pre- and post- balloon pulmonary angioplasty (BPA) treatment in 8 patients.

### RHC measurements

RHC was measured after stabilization of the hemodynamics, which included mean pulmonary arterial pressure (mPAP), pulmonary artery wedge pressure (PAwP), cardiac output (CO), cardiac index (CI), and pulmonary vascular resistance (PVR). Precapillary PH was retained if PAwP was ≤ 15 mmHg, and postcapillary PH was retained if PAwP was > 15 mmHg.

### CT performance

Imaging analysis was based on the observation of contrast-enhanced HRCT of the chest or CTPA. The stenosis in pulmonary, tracheal, bronchial and esophageal within the mediastinum was evaluated. Pulmonary effusion, lymph node calcification, atelectasis, and pleural thickening were evaluated as well.

### Statistical analysis

Continuous variables with normal distribution are presented as mean value ± standard deviation, whereas those with skewed distribution are presented as median and interquartile range. Differences between groups were tested using Student’s t-test or the rank-sum test. Categorized data are presented as numbers and proportions, which were formally evaluated with the Chi-square test or Fisher exact test. Paired samples t-tests were used for comparison of parameters pre- and post- BPA treatment. Two-tailed P-values < 0.05 were considered to be statistically significant. All statistical analyses were performed using SPSS version 25.

## Results

### Clinical manifestations and examinations

The clinical characteristics of the 51 patients included in the study are shown in Table 1. The proportion of female patients is higher (SPAP < 50 mmHg group, M/F = 8/18; SPAP ≥ 50 mmHg group, M/F = 7/18). The most common symptoms included dyspnea in 48 patients (94.1%), cough in 40 patients (78.4%), and expectoration in 36 patients (70.6%). No patient was found to have clinical superior vena cava syndrome or hoarseness. Elevated CRP were obtained for 12 patients (23.5%). Elevated ESR measurements were obtained for 9 patients (17.6%). Among 27 patients (52.9%) who were confirmed to have a history of tuberculosis (TB), 17 patients had received standard treatment for TB before the diagnosis of FM. The etiology was undetermined in all other patients included in the study. Among the 11 patients who received RHC, the median mPAP was 40.00 (interquartile range: 29.00 to 45.00) mmHg; the median PAwP was 8 (interquartile range: 7.00 to 8.00) mmHg; the median CO was 3.98 (interquartile range: 3.83 to 4.75) L/min; the median CI was 2.75 (interquartile range: 2.19 to 3.30) L/min/m^2^; and the median PVR was 6.95 (interquartile range: 4.64 to 8.74) Wood units. Precapillary PH was noted in all patients who underwent RHC. Among the 51 patients included in the study, 27 (52.9%) underwent bronchoscopy, and 22 (22/27, 81.5%) had bronchial stenosis. Diffuse black pigmentation in the bronchial mucosa was found in 17 patients (17/27, 63.0%); intra-tracheal hemorrhage and necrosis were each found in one patient.

**Table 1:** Clinical manifestations and characteristics of patients included in the study

	FM (n = 51)	Group of FM		P value
		SPAP < 50 mmHg group; n = 26	SPAP ≥ 50 mmHg groupn = 25	
**General information**				
Age at time of diagnosis, yrs	64.04 ± 11.46	66.42 ± 9.99	61.56 ± 12.52	0.131
Male, %	15(29.4%)	8(30.8%)	7(28.0%)	0.828
History of smoking	12(23.5%)	8(30.8%)	4(16.0%)	0.214
**Tuberculosis**				
Confirmed	27(52.9%)			
Suspected	7(13.7%)			
**Clinical presentation**				
Dyspnea	48(94.1%)	23(88.5%)	25(100.0%)	0.235
Cough	40(78.4%)	20(76.9%)	20(80.0%)	0.789
Expectoration	36(70.6%)	17(65.4%)	19(76.0%)	0.406
Hemoptysis	9(17.6%)	6(23.1%)	3(12.0%)	0.465
Chest pain	6(11.8%)	4(15.4%)	2(8.0%)	0.668
Chest tightness	25(49.0%)	10(38.5%)	15(60.0%)	0.124
Palpitation	1(2.0%)	0(0.0%)	1(4.0%)	0.490
Fever	9(17.6%)	5(19.2%)	4(16.0%)	0.762
Peripheral edema	19(37.3%)	10(38.5%)	9(36.0%)	0.856
SVCS	0(0.0%)	0(0.0%)	0(0.0%)	–
Hoarseness	0(0.0%)	0(0.0%)	0(0.0%)	–
CRP	12(23.5%)	7(26.9%)	5(20.0%)	0.560
ESR	9(17.6%)	7(26.9%)	2(8.0%)	0.140

SVCS = superior vena cava syndrome; ESR = erythrocyte sedimentation rate; CRP = c-reactive protein

### Echocardiography

**Fig. 1:**
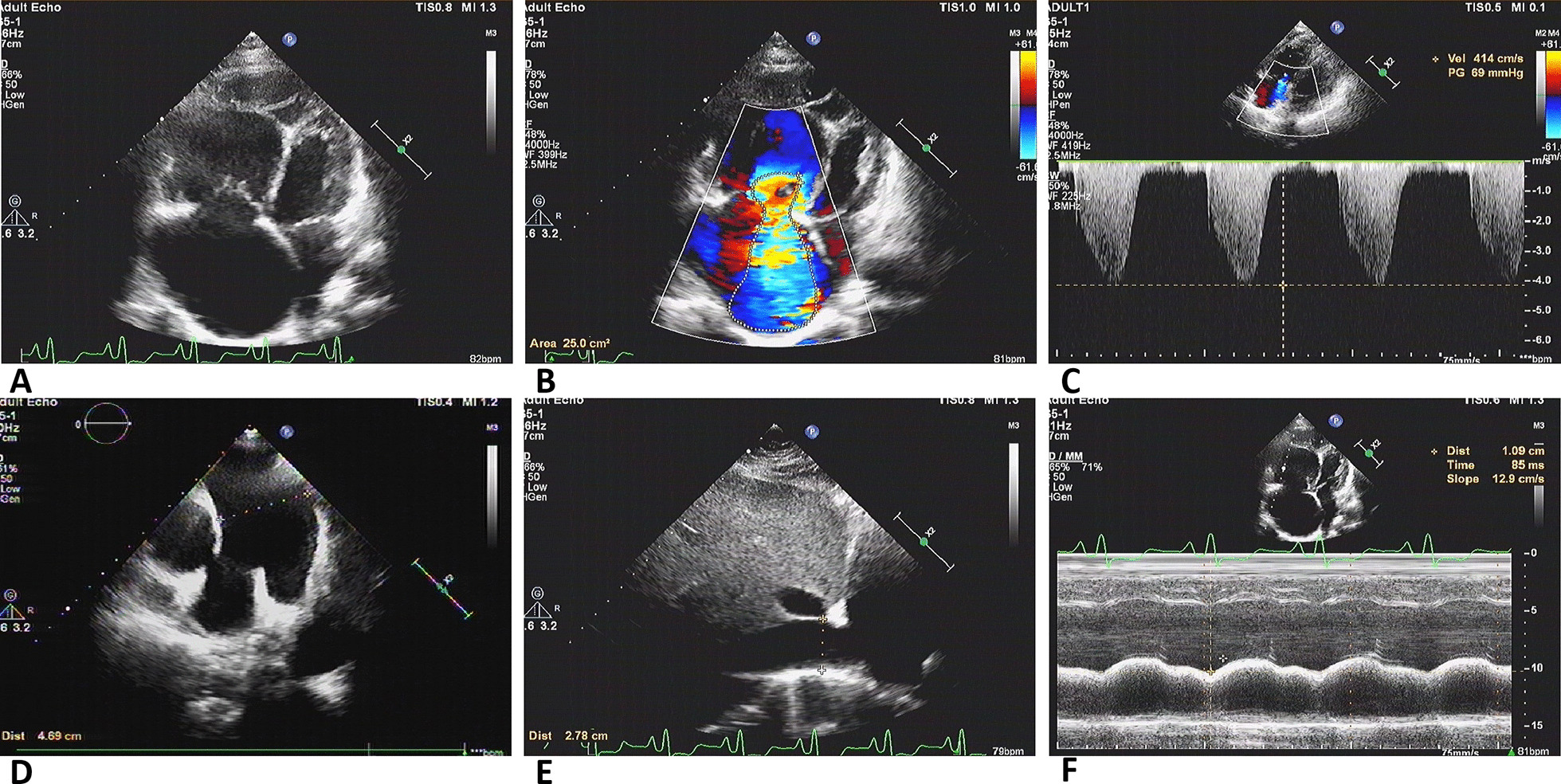
Echocardiography performed on patients with fibrosing mediastinitis complicated with pulmonary hypertension. **a** Echocardiography revealed an increase in right heart ratio. **b** Severe tricuspid regurgitation. **c** Tricuspid regurgitation with a Vel of 414 cm/sec and a PG of 69 mmHg. **d** Enlarged diameter of the main pulmonary artery (D_MPA_). **e** Enlarged diameter of the inferior vena cava (D_SVC_). **f** Decrease of approximately 10.9 mm in tricuspid annular plane systolic excursion (TAPSE)

All 51 patients underwent echocardiography at least once during hospitalization. The results, stratified according to the level of SPAP, are shown in Table 2. Right heart diameter was greater in the SPAP ≥ 50 mmHg group. RV basal diameter (p = 0.012) and inner diameter of the right atria (p = 0.011) differed significantly between groups. And differences between groups were detected for RA/LA (SPAP < 50 mmHg group, mean < 1; SPAP ≥ 50 mmHg group, mean > 1; *p* < 0.01) and RV/LV (SPAP < 50 mmHg group, mean < 1; SPAP ≥ 50 mmHg group, mean > 1; *p* < 0.01). In addition, V_TR_ values were significantly greater among patients in SPAP ≥ 50 mmHg group, compared with patients in SPAP < 50 mmHg group (412.08 ± 56.18 cm/sec vs. 288.76 ± 33.90 cm/sec, *p* < 0.001). Neither D_MPA_ nor LVEF differed between groups. The data pertaining to RV function showed that more than half of the patients in SPAP ≥ 50 mmHg group (14 of 25, 56.0%) had reduced RV function to a varying degree. All patients in SPAP < 50 mmHg group had normal RV function. The manifestations on echocardiography are shown in Fig. 1. In many FM patients with PH, echocardiography results revealed an enlarged right heart, tricuspid regurgitation, widening of the main pulmonary artery, and widening of the inferior vena cava.

**Table 2:** Echocardiographic parameters in patients with SPAP < 50 mmHg vs. SPAP ≥ 50 mmHg

	Group of FM		P value
	SPAP < 50 mmHg group n = 26	SPAP ≥ 50 mmHg group n = 25	
RA, mm	32.28 ± 3.91	36.72 ± 7.33	0.011
RA/LA	0.93 ± 0.08	1.23 ± 0.40	0.001
RV, mm	31.82 ± 3.62	36.90 ± 8.90	0.012
RV/LV	0.93 ± 0.13	1.12 ± 0.23	0.002
D_MPA,_ mm	26.43 ± 6.06	29.56 ± 6.40	0.079
V_TR_, cm/sec	288.76 ± 33.90	412.08 ± 56.18	< 0.001
LVEF, %	70.22 ± 5.89	68.20 ± 7.87	0.304

RA = right atrium transverse dimension in four-chamber apical view; RA/LA = right atrium transverse dimension/left atrium transverse dimension in four-chamber apical view; RV = basal right ventricular linear dimension in four-chamber apical view; RV/LV = basal right ventricular linear dimension/basal left ventricular linear dimension in four-chamber apical view; D_MPA_ = diameter of main pulmonary artery; V_TR_ = velocity of tricuspid regurgitation; LVEF = left ventricular ejection fraction

### Functional characteristics

A total of 34 patients underwent pulmonary function testing. The results showed an obstructive pattern in 21 patients, a restrictive pattern in 3 patients, and a mixed pattern in 2 patients, and 8 patients had normal lung volumes and flows. Median values for FEV1%pred, FEV1/FVC, TLC%pred, and DLCO%pred were smaller in patients from SPAP ≥ 50 mmHg group, compared with patients from the SPAP < 50 mmHg group. However, these differences did not reach statistical significance (Table 3). Arterial blood gas abnormalities were common, but no significant difference was observed between patients after stratification by SPAP.

**Table 3:** Clinical characteristics in patients with SPAP < 50 mmHg vs. SPAP ≥ 50 mmHg

	FM group		P value
	SPAP < 50 mmHg group n = 26	SPAP ≥ 50 mmHg group n = 25	
**Anatomic distribution of FM within the mediastinum**			
Bilateral	18(69.2%)	20(80.0%)	0.378
Unilateral	0(0.0%)	0(0.0%)	–
Diffuse	8(30.8%)	5(20.0%)	0.378
Pulmonary arteries compression	19(73.1%)	23(92.0%)	0.140
Main pulmonary arteries	3(11.5%)	4(16.0%)	0.703
Lobar arteries	18(69.2%)	22(88.0%)	0.103
Pulmonary veins compression	5(19.2%)	7(28.0%)	0.460
Superior vena cava compression	0(0.0%)	0(0.0%)	–
Bronchial compression	11(42.3%)	9(36.0%)	0.645
Esophagus compression	1(3.8%)	1(4.0%)	1.000
Others			
Pleural effusion	5(19.2%)	12(48.0%)	0.029
Pericardial effusion	2(7.7%)	5(20.0%)	0.248
Calcification of Lymph node	10(38.5%)	13(52.0%)	0.331
Pleural thickening	15(57.7%)	19(76.0%)	0.166
Segmental or subsegmental atelectasis	5(19.2%)	10(40.0%)	0.104
**Arterial blood gas analysis**			
PaO2, kpa	9.83 ± 2.12	9.26 ± 1.70	0.321
PaCO2, kpa	5.24 ± 0.85	5.41 ± 1.35	0.608
SaO2, %	95.0(91.8,97.0)	94.2(91.0,95.6)	0.211
**Pulmonary function, n = 34**	**n = 18**	**n = 16**	
FVC, %pred	94.15(80.70,111.73)	95.70(71.28,103.20)	0.506
FEV1, %pred	70.95(58.48,98.53)	66.70(51.85,79.78)	0.224
FEV1/FVC	63.63(55.02,75.66)	61.37(56.90,70.35)	0.670
TLC, %pred	93.45(85.35,101.20)	92.25(77.48,97.75)	0.646
DLCO, %pred	71.65(64.25,81.93)	63.30(47.23,77.65)	0.081

% pred = percent of predicted value; PaO2 = partial pressure of oxygen; PaCO2 = partial pressure of carbon dioxide; SaO2 = oxygen saturation; FVC = forced vital capacity; FEV1 = forced expiratory value in 1 s; TLC = total lung capacity; DLCO = diffusion capacity of carbon monoxide

### Radiologic findings

HRCT and CTPA were performed at the time of the initial evaluation for all patients included in the study. The differences in radiological features between groups are shown in Table 3. Anatomic distributions of FM within the mediastinum were evident on CT in all 51 patients. The results showed that FM patients were prone to pulmonary artery stenosis (42/51, 82.4%), especially stenosis affecting the lobar pulmonary artery. Mediastinal lymph node calcification (45.1%) and pleural thickening (66.7%) were also common, while the prevalence of pleural effusion was higher in SPAP ≥ 50 mmHg group than in SPAP < 50 mmHg group (p = 0.029).

### Treatments

**Fig. 2:**
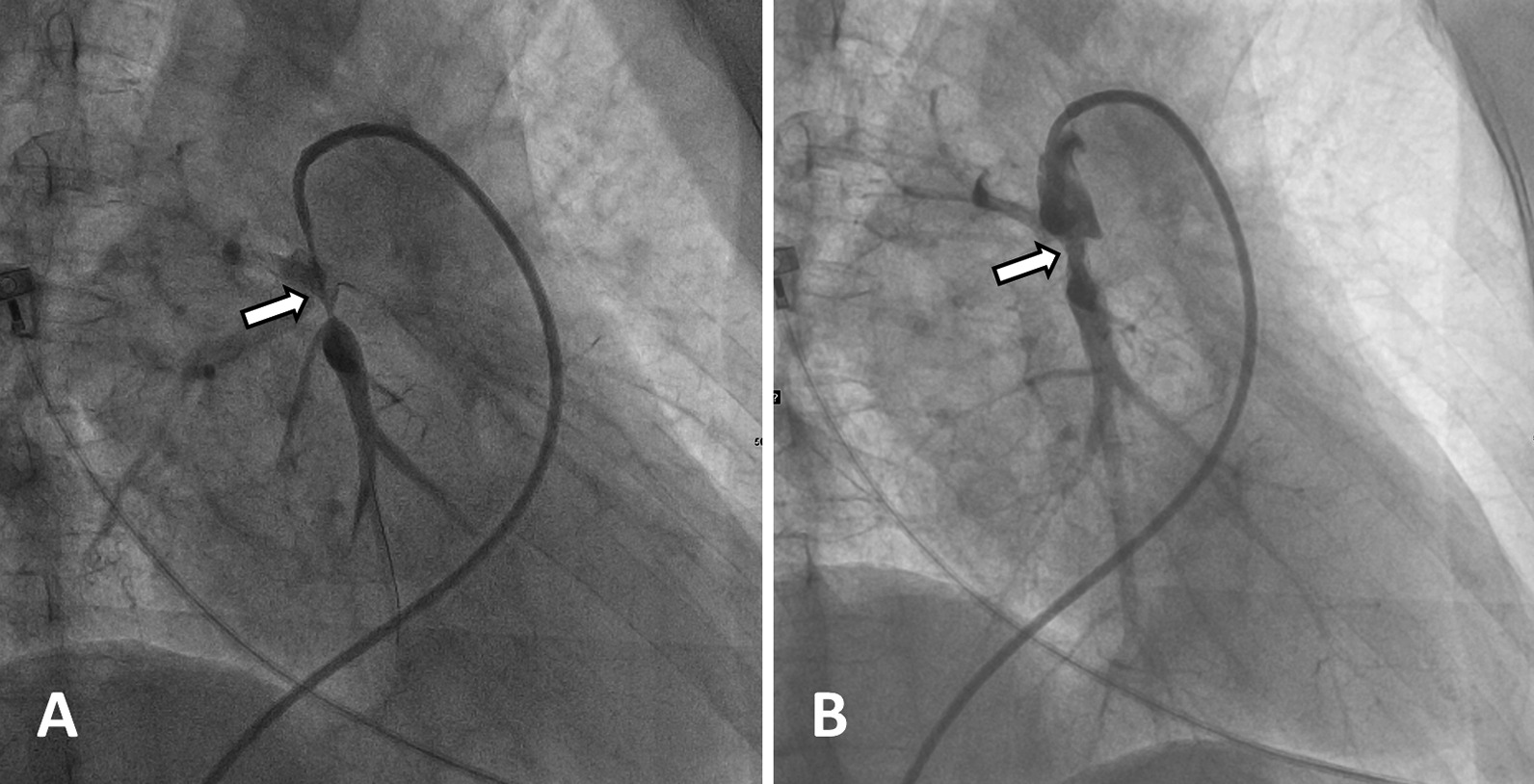
DSA images of the pulmonary artery in a patient with FM and PH that received BPA. **a** The arrow points to vascular stenosis in an image obtained before balloon pulmonary angioplasty (BPA). **b** The arrow points to the position of the endovascular stent after BPA treatment

Most patients in this study received medical treatments only. Eight patients underwent BPA (1 to 5 times), four of whom underwent endovascular stent implantation. The changes in echocardiography measurements pre- and post- BPA are shown in Table 4. Images obtained perioperatively are shown in Fig. 2. After treatment, the proportion of atrial volume in the right atrium decreased significantly (1.05 ± 0.13 vs. 0.91 ± 0.04, p = 0.007) (Additional file 1: Table S1). Differences in inner diameter of the right heart chamber and SPAP after BPA were not statistically significant. However, SPAP values were decreased postoperatively in 5 patients who underwent BPA (Table 4).

**Table 4:** Changes in echocardiographic parameters pre- and post-BPA

	ΔRA, mm	ΔRA/LA	ΔRV, mm	ΔRV/LV	ΔSPAP, mmHg	ΔD_MPA_, mm
1	− 2.00	0.04	− 2.00	0.02	− 4.50	− 3.00
2	4.00	0.08	1.00	0.08	− 4.00	− 3.00
3	4.00	0.35	1.00	0.06	5.80	4.00
4	5.00	0.11	− 8.00	0.24	4.50	0.00
5	7.00	0.13	3.00	− 0.03	29.00	7.00
6	0.00	0.24	5.00	0.07	24.10	− 3.00
7	− 1.00	0.02	8.00	0.17	20.40	− 0.50
8	5.00	0.17	2.00	− 0.22	− 2.00	− 1.00

Δ Indicates the difference between pre- vs. post-BPA. RA = right atrium transverse dimension in four-chamber apical view; RA/LA = right atrium transverse dimension/left atrium transverse dimension in four-chamber apical view; RV = basal right ventricular linear dimension in four-chamber apical view; RV/LV = basal right ventricular linear dimension/basal left ventricular linear dimension in four-chamber apical view, SPAP = systolic pulmonary artery pressure; D_MPA_ = diameter of main pulmonary artery; LVEF = left ventricular ejection fraction

## Discussion

The major findings of the present study include: (1) FM patients with increased SPAP, as measured by echocardiography, may have more adverse changes in cardiac chamber structure and function, especially in the right heart; (2) CT findings may be insufficient to distinguish different hemodynamic states in patients with FM; and (3) echocardiography is valuable in the management of FM, particularly for those with PH, in view of its advantages in evaluating cardiac structure, function and hemodynamics.

In this study, FM was more common in older and female patients. The pathogenesis of FM remains to be elucidated. A delayed hypersensitivity reaction caused by fungal or mycobacterium infection [12, 13], an autoimmune process (such as Behçet’s disease, rheumatoid arthritis, or systemic lupus erythematosus) have been proposed as the potential underlying mechanisms[13–15]. Clinically, histoplasmosis is considered to be the most common cause of FM, while tuberculosis-related FM is considered to be a common cause of FM in specific areas, such as China [16–18]. More than half of the patients in this study had FM associated with tuberculosis. Common symptoms included dyspnea, cough, and expectoration, and nearly 50% of patients had chest tightness and peripheral edema. The occurrence of these symptoms may be related to the stenosis of pulmonary vessels and the bronchial tree. In a small number of patients, lesions may affect the esophagus, mediastinal nerve, and even the heart, resulting in dysphagia and hoarseness [19]. However, there was no significant difference in symptoms among patients with different SPAP in this study.

PH caused by FM is mainly related to compression of the pulmonary artery in FM, which is classified as Group 5 PH [6] and may cause progressive right heart dysfunction. This study showed that patients in FM with significant increased SPAP by echocardiography do have adverse structural and functional changes of the right heart. According to the guidelines, PH is diagnosed in the presence of mPAP ≥ 25 mmHg, as measured during RHC at sea level, with the patient at rest [20]. RHC remains the primary standard for the evaluation of PH. However, only a small proportion of patients (n = 11) in this study received RHC due to the invasiveness of the examination.

The results of echocardiography may be useful in the preliminary assessment of pulmonary artery pressure because of the convenience and noninvasiveness of the examination. Analysis of the results of echocardiography showed that the right heart is larger in patients with SPAP ≥ 50 mmHg than in patients with SPAP < 50 mmHg. Most of the FM patients with SPAP ≥ 50 mmHg had an increase in proportional volume of the right heart, which may be related to an increase in afterload. In the meantime, they are more likely to have decreased right heart function, perhaps because of long-term changes in hemodynamics and heart structure. It has been reported that echocardiography can detect the high-velocity continuous jets in the left atrium that originate from the right and left major pulmonary veins in patients with pulmonary vein stenosis caused by FM [21]. The reduction in pulmonary vein diameter and increases in blood flow velocity and turbulence visualized with color-flow Doppler may be helpful for assessing involvement of the main pulmonary veins. In our study, patients were found to have various types of respiratory dysfunction, which can lead to abnormal blood gas values and exercise intolerance. Besides, no significant difference in these measurements was found between patients with different SPAP, indicating the limited value of these parameters for the detection of abnormal hemodynamics in patients with FM. To sum up, echocardiography can easily be applied to evaluate increased pulmonary artery pressure and possible changes in right heart structure and function in FM, which is helpful for decision making during clinical management.

Generally, a diagnosis of FM requires positive findings on HRCT or CTPA in combination with clinical manifestations. Imaging studies have shown that patients with FM usually have a diffuse, invasive, or well-defined mediastinal mass, leading to local stenosis or secondary dilation of pulmonary, tracheal, or bronchial vessels. Some studies have shown that, compared to FM caused by histoplasmosis infection, FM caused by *Mycobacterium tuberculosis* (MTB) is more likely to present with stenosis of the above structures [17]. Half of the patients included in our study had a clear history of tuberculosis, and the results of radiologic examinations were indeed consistent with the above findings. Although CT has definite advantages in revealing of stenotic structures, the evaluation of abnormal hemodynamics and secondary cardiac changes may not be better than echocardiography. There is no significant difference between FM patients with SPAP < 50 mmHg and SPAP ≥ 50 mmHg in terms of the anatomy involved, except for the presence of pleural effusion. Transudatory hydrothorax caused by increased pulmonary vascular pressure may be the reason that FM patients with SPAP ≥ 50 mmHg are more likely to have pleural effusion. In addition, some studies have suggested that pericardial effusion may predict poor prognosis in patients with group 5 PH [6].

Treatments for FM are currently limited, which is one of the reasons for the poor prognosis of patients. Medications such as antifungal therapy, antituberculosis therapy, and glucocorticoids may stabilize disease progression or lead to limited symptomatic improvement [5]. In addition, the use of specific PH therapy in FM patients with PH may be not ideal at present [6]. After a comprehensive assessment of the patient's condition, BPA, intravascular or endobronchial stent placement and surgical intervention are usually performed to relieve stenosis in bronchi or vessels [7, 22]. However, most of the relevant valid data is based on case reports or small case series. In this study, 8 patients were treated with BPA. Serial echocardiographic measurements in these patients showed that only the volumetric proportion of the right atrium decreased significantly. Other changes in echocardiographic measurements were all not remarkable. Given the heterogeneity of clinical characteristics in patients with FM (location of the lesion, degree of stenosis, growth characteristics of the lesion), the efficacy of BPA may vary. When etiology remains unknown, restenosis is likely to occur [8, 23]. Although the sample size was limited, SPAP values improved in 5 of the 8 patients after treatment, suggesting that BPA may be a promising treatment for improving hemodynamics in FM patients with PH. To make the appropriate adjustments to the treatment plan in an expedient fashion, clinicians must regularly monitor the patient’s clinical symptoms, lesion progression, pulmonary artery pressure, cardiac structure, and cardiac function. The echocardiographic measurements obtained for this study illustrated the effects of a single BPA. Additional studies with larger sample size and detailed follow-up information are needed. As a noninvasive, simple, and relatively low-cost diagnostic tool, echocardiography may helpful in the elaboration of a treatment plan and the evaluation of curative effects, especially for FM patients with elevated SPAP.

Our study has several limitations. First, it is a retrospective study in which the integrity of the clinical data for some patients was suboptimal, and long-term follow-up results are lacking. Because of the lack of quantitative data, right ventricular dysfunction is defined as a reduction with one or more than one functional parameter. Secondly, due to the rarity of the disease, the number of cases included in our study was limited. The patient population was relatively homogeneous, in terms of ethnicity and geographic region of residence. The diagnosis of FM was confirmed by HRCT or CTPA rather than histological analysis because biopsies require mediastinoscopy or endoscopic bronchial ultrasound fiberoptic bronchoscopy, both of which are high-risk procedures. RHC measurements, which are the gold standard for assessing pulmonary artery pressure, were insufficient in this study. Although there are some biases in the assessment of SPAP by echocardiography, especially for patients with significant stenosis of the proximal pulmonary artery, which can lead to an overestimation of SPAP. However, referring to the guidelines, we grouped according to the higher SPAP level, which indicates a high risk of PH. There were only 4 patients with main pulmonary artery involvement in SPAP ≥ 50 mmHg group, and the main pulmonary artery stenosis was not significant in CT. Furthermore, existing research shows that echocardiographic measurements of SPAP have good accuracy in the evaluation of PH. So it is considered that the bias may not have a significant impact on our conclusions. Due to the retrospective nature of the study, there is a lack of more quantitative information on the segments and degree of stenosis of the vessels on CT, which should be paid more attention to in further research. Several patients who had received BPA for preliminary evaluation of curative efficacy were included. However, this study only collected the echocardiographic measurements of BPA for one time. Comprehensive clinical evaluation and long-term follow-up data were missing. Therefore, further research is meaningful and necessary for BPA in patients with FM. Meanwhile, etiological factors and restenosis need to be noticed.

In conclusion, PH is one of the important complications in patients with FM and has been associated with poor prognosis. Significant changes in right heart structure and function caused by elevated pulmonary arterial pressure may be observed. Although CT is valuable in the detection of lesions and diagnosis of FM, echocardiography is more helpful to reveal the abnormal hemodynamic state and the changes of cardiac structure and function, which may provide further information for the determination of prognosis. Echocardiography is necessary for clinical management of the patient, especially for those with elevated SPAP as measured by echocardiography, in the determination of appropriate treatments.

## Supplementary Information

**Additional file 1. Supplemental Table 1.** Echocardiographic parameters pre-BPA vs. post-BPA.

## Data Availability

The datasets generated and analyzed during the current study are not publicly available due to the restrictions by the Beijing Chaoyang Hospital. The authors used this dataset under an agreement with the Beijing Chaoyang Hospital for the present study. The data are available from the corresponding author on reasonable request.

## Abbreviations

BPA: Balloon pulmonary angioplasty
CTEPH: Chronic thromboembolic pulmonary hypertension
CI: Cardiac index
CO: Cardiac output
CRP: C-reactive protein
CT: Computed tomography
CTPA: Computed tomography pulmonary angiography
DLCO: Lung diffusing capacity for carbon monoxide
DMPA: Diameter of the main pulmonary artery
DSVC: Internal diameter of the inferior vena cava
ESR: Erythrocyte sedimentation rate
FM: Fibrosing mediastinitis
FVC: The forced vital capacity
FEV1: The forced expiratory volume in 1s
HRCT: High-resolution computed tomography
LVEF: Left ventricular ejection fraction
MTB: Mycobacterium tuberculosis
mPAP: Mean pulmonary arterial pressure
PAwP: Pulmonary artery wedge pressure
PH: Pulmonary hypertension
PVR: Pulmonary vascular resistance
RAP: Right atrial pressure
RHC: Right-sided heart catheterization
RIMP: Right ventricular index of myocardial performance
RV: Right ventricle/ventricular
RVFAC: Right ventricular fractional area change
PaO2: The arterial partial pressure of oxygen
PaCO2: The partial pressure of carbon dioxide
SaO2: Oxygen saturation
SPAP: Systolic pulmonary arterial pressure
TAPSE: Tricuspid annular plane systolic excursion
TLC: Total lung capacity
VTR: Velocity of tricuspid regurgitation
6MWD: 6-Minute walk distance
