# Supplementary material for: Comparison of fibrosing mediastinitis patients with vs. without markedly increased systolic pulmonary arterial pressure: a single-center retrospective study
Source: BMC Cardiovasc Disord. 2022 Mar 31;22:134. doi: 10.1186/s12872-022-02567-z (PMC8973553; doi:10.1186/s12872-022-02567-z)
Supplement: Supplementary file 1 — Additional file 1. Supplemental Table 1. Echocardiographic parameters pre-BPA vs. post-BPA. [file 12872_2022_2567_MOESM1_ESM.doc]

**Supplemental Table 1. Echocardiographic parameters pre-BPA vs. post-BPA**

|  | BPA Group | |  |
| --- | --- | --- | --- |
|  | Pre-BPA  (n=8) | Post-BPA  (n=8) | P value |
| RA, mm | 35.13±2.85 | 32.38±3.62 | 0.050 |
| RA/LA | 1.05±0.13 | 0.91±0.04 | 0.007 |
| RV, mm | 35.25±4.10 | 34.00±3.16 | 0.483 |
| RV/LV | 0.86±0.09 | 0.81±0.13 | 0.341 |
| SPAP, mmHg | 53.55±20.40 | 44.39±17.60 | 0.095 |
| D_MPA_, mm | 29.13±6.27 | 29.06±5.21 | 0.963 |

RA=right atrium transverse dimension in four-chamber apical view; RA/LA=right atrium transverse dimension/left atrium transverse dimension in four-chamber apical view; RV=basal right ventricular linear dimension in four-chamber apical view; RV/LV=basal right ventricular linear dimension/basal left ventricular linear dimension in four-chamber apical view, SPAP=systolic pulmonary artery pressure; D_MPA_=diameter of main pulmonary artery.
